# Modulation of apoptosis by V protein mumps virus

**DOI:** 10.1186/1743-422X-8-224

**Published:** 2011-05-13

**Authors:** Nora H Rosas-Murrieta, Gerardo Santos-López, Julio Reyes-Leyva, Francisca Sosa Jurado, Irma Herrera-Camacho

**Affiliations:** 1 Laboratorio de Bioquímica y Biología Molecular, Instituto de Ciencias, Benemérita Universidad Autónoma de Puebla, Edif. 103H, CU-BUAP, San Manuel, CP 72550, Puebla, México; 2 Laboratorio de Virología, Centro de Investigación Biomédica de Oriente, Instituto Mexicano del Seguro Social, Km 4.5 carretera Atlixco-Metepec, CP 74360 Metepec, Puebla, México

## Abstract

**Background:**

The Urabe AM9 vaccine strain of mumps virus contains two variants of V protein: VWT (of HN-A1081 viral population) and VGly (of HN-G1081). The V protein is a promoting factor of viral replication by blocking the IFN antiviral pathway.

**Findings:**

We studied the relationship between V protein variants and IFN-α2b-induced apoptosis. V proteins decrease activation of the extrinsic IFN-α2b-induced apoptotic pathway monitored by the caspase 8 activity, being the effect greater with the VWT protein. Both V proteins decrease the activity of caspase 9 of the intrinsic apoptotic pathway. In a system without IFN, the VWT and VGly proteins expression promotes activation of caspases 3 and 7. However, when the cellular system was stimulated with IFN-α, this activity decreased partially. TUNEL assay shows that for treatment with IFN-α and ibuprofen of cervical adenocarcinoma cells there is nuclear DNA fragmentation but the V protein expression reduces this process.

**Conclusions:**

The reduction in the levels of caspases and DNA fragmentation, suggesting that V protein, particularly VWT protein of Urabe AM9 vaccine strain, modulates apoptosis. In addition, the VWT protein shows a protective role for cell proliferation in the presence of antiproliferative signals.

## Findings

The IFN type-I pathway is the major cellular mechanism of the antiviral response. The effect is the induction of gene expression to blocking the viral infection. The efficient antiviral cellular response promotes the development of viral strategies to antagonize the effect of IFN [1-4]. In the paramyxoviruses, inhibition of IFN type-I response occurs due to the activity of the nonstructural V protein [5,6]. Activation of the JAK-STAT pathway by IFN simultaneously activates others processes regulated by IFN such as apoptosis, a physiological process where cells undergo morphological changes, activation of proteases, nuclear DNA fragmentation and cell death [1,2]. The central component of the apoptotic machinery is a proteolytic system consisting of the family of cysteine proteases (caspases) [7-9]. Apoptosis can be initiated and executed through many different pathways, which can be categorized into two main groups: extrinsic and intrinsic [8,9]. Sequential activation of a caspase by another creates an expansive cascade of proteolytic activity that produces digestion of structural proteins in the cytoplasm, DNA degradation and phagocytosis of apoptotic bodies [10]. Because apoptotic cells are rapidly phagocytosed, apoptosis promotes development of an efficient immune response against viral antigens [11]. Many viruses have evolved mechanisms to avoid or at least to control apoptosis [12]. One of the mechanisms of pathogenicity of mumps virus is V protein expression required to blocking the expression of viral genes activated by IFN through cytoplasmic interaction with the STAT1-STAT2 heterodimer [13,14]. What cellular process is the prime target to promote viral replication? Effects of the inactivation of the IFN pathway on apoptosis, in particular, are not known in detail. IFN-α induces apoptosis and stimulates the activity of caspases 1, 2, 3, 8 and 9 and promotes the extrinsic apoptotic pathway and the activation of caspase 8 as the initiator of the caspase cascade to execute apoptosis [15].

In this study we analyzed the effect of the VWT **(**from the HN-A1081 population, neurovirulence associated) and VGly (from the HN-G1081 population) proteins of the Urabe AM9 strain vaccine of mumps virus [16-18], in order to determine whether, as the simian virus 5 V protein [19] and the C protein parainfluenza virus type I (HPIV-1) [20], they have the capacity of blocking IFN-α-induced apoptosis. VWT and VGly of the Urabe AM9 strain were expressed in cervical adenocarcinoma cells to analyze its effect on the activity of initiator and effector caspases. The cells were transfected with 10 μg of vector DNA (pCDNA4/His/Max-VA and VG) and TurboFect transfection reagent (Fermentas, Glen Burnie, MD, USA). 36 h after transfection, the cells were treated with 4000 IU/mL of IFN-α2b (Urifrón, Probiomed, Mexico) and 40 μM of MG-132 (Sigma, St. Louis, MO, USA). The activity of caspases 3, 7 and 8 was evaluated with the Caspase-Glo 3/7 kit and Caspase-Glo 8 kit (Promega, Madison, WI, USA) using the substrate Ac-DEVD-pNA for caspase 3/7 or a C-LETD-pNA for caspase 8 and incubated for 60 min at room temperature. The activity of caspases 3, 7 and 8 was measured by a GloMax 20/20 luminometer (Promega, Madison, WI, USA). The activity of caspase 9 was measured with the Caspase 9 Colorimetric Assay Kit (Biovision) using LEDH-pNA as substrate by absorbance at 420 nm, 2 h after adding substrate.

**Figure 1:**
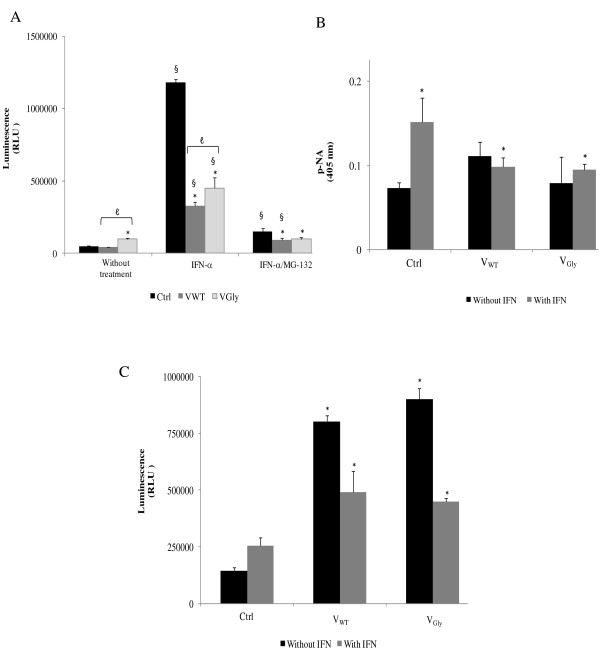
**Activity of caspase 8, caspase 9 and caspase 3/7 in the cervical carcinoma cell line expressing VWT and VGly proteins of mumps virus**. (A) Caspase 8 measured with the Caspase-Glo 8 assay (Promega) using Ac-LETD-pNA as substrate. *, statistical difference between samples treated with IFN and IFN/MG132 with respect to untreated group. §, statistical difference between samples with and without treatment with IFN and IFN/MG132. ℓ, statistical difference between samples with VWT and VGly. (B) Caspase-9 measured with the colorimetric assay Caspase 9 (BioVision) using LEDH-pNA as substrate. The absorbance was taken at 420 nm in a Shimadzu spectrophotometer 2 h after adding substrate. (C) Caspase 3 and 7 measured with the Caspase-Glo 3/7 assay system (Promega) using the substrate Ac-DEVD-pNA. Readings were taken 1 h after in the GloMax 20/20 luminometer (Promega). Each column represents the mean ± SD of three independent experiments. (In B and C, * represents the statistical difference between samples with and without IFN). Student t-test (*P *< 0.05).

This study starts with the analysis of the effect of V proteins on caspase 8 activity. In the system without IFN-α, low activity of caspase 8 was detected, although expression of VGly increases the activity in 113% (Figure 1A). Instead, the treatment of the system with IFN-α2b promotes an increase of 2400% of the enzymatic activity in control cells, which decreases in those that express VWT and VGly in 73% and 38%, respectively. The decrease was greater with the VWT protein. The same activity pattern was recorded in cells stimulated with IFN-α2b and with the MG132 proteasome inhibitor. In control cells the activity increased by 224%, whereas that of VWT and VGly increased by only 96% and 113%, respectively, without significant difference in the decrease of caspase 8 activity (Figure 1A). We can conclude the VWT protein of Urabe AM9 strain has a greater inhibitory effect on the activity, i.e., the protein derived from the HN-A1081 population associated with neurovirulence may modulate the extension in the activation of the extrinsic apoptotic pathway.

On the other hand, cytoplasmic replication of mumps virus produces dsRNA molecules that may activate the intrinsic apoptotic pathway by intracellular stress and the V proteins of mumps virus may have an effect on apoptosis that involves the intrinsic or mitochondrial pathway. In the system without IFN-α2b treatment, it was demonstrated that the enzyme activity increased 51% in cells that express the VWT and 8% in cells with VGly (Figure 1B). Treatment of the C33 cell line with IFN-α2b activates the mitochondrial apoptotic pathway detected by an increase of 107% in caspase 9 activity. In contrast, in the IFN-treated cells that express VWT and VGly proteins, the activity of caspase 9 decreased 35% and 38%, respectively (Figure 1B), suggesting the activation of the apoptotic mitochondrial pathway, which may be reduced by expression of V proteins of the mumps virus regulating the magnitude of apoptosis. Although data are obtained for detection of caspase 9, a high activation is not observed as with caspase 8. If there is strong activation of the intrinsic apoptotic pathway by another factor of mumps virus in combination with the V protein, this must be analyzed in further studies.

Caspases 8 and 9 are directly responsible for activation of the effector caspases 3 and 7. Their activity was analyzed in cells non-transfected and without stimulation with IFN-α2b, where we found that there is a low level of enzymatic activation probably due to the introduction of DNA plasmid (pCDNA4/HisMaX), whereas in cells that express VWT and VGly proteins there is an increase in the activity of caspase 3 and 7 of 457% and 526%, respectively (Figure 1C). When the system was stimulated with IFN-α2b, the activity of caspases 3 and 7 increased 77%, 91% y 76% in control cells, and with the VWT and VGly proteins, respectively, compared with to the system without treatment. A detailed comparison of caspases 3 and 7 activity with or without IFN shows that V proteins partially inhibit the activity, although there was no statistically significant difference on enzymatic activity in both proteins. These data suggest a partial inhibitory activity of 61% and 49%, respectively, on the IFN-α-induced apoptosis by the VWT and VGly proteins of mumps virus (Figure 1C). Despite activation of the IFN pathway, activity of caspases 3 and 7 decreased compared to cells that did not express the viral protein, suggesting a role for the V protein in the disruption of the antiviral response controlling the apoptosis. However, V proteins of Urabe AM9 vaccine strain did not completely inhibit caspase activity in our study and, therefore, the apoptosis. Despite the partial inhibition of caspases 8, 3 and 7, the reported residual activity suggests that nuclear DNA fragmentation, a key process in apoptosis, should be carried out.

**Figure 2:**
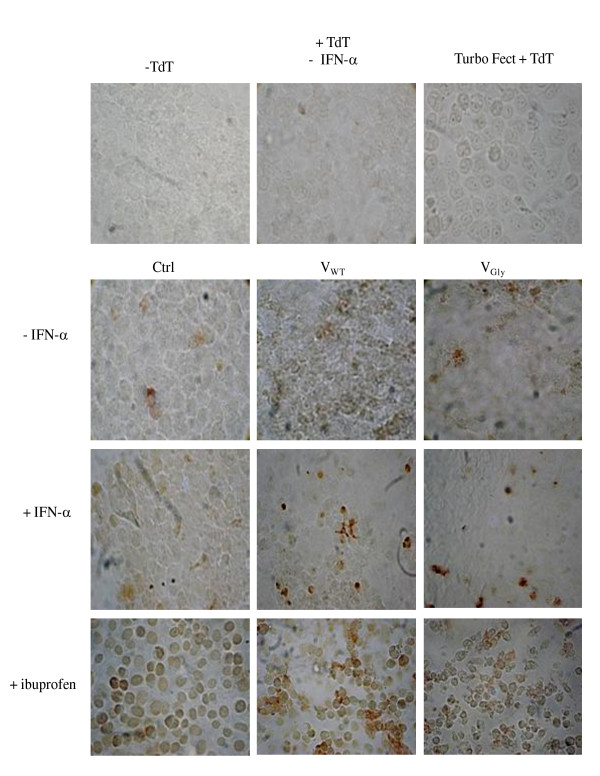
**Apoptosis in cervical adenocarcinoma cells that express proteins VWT and VGly**. At 48 h of expression, 2 h with 4000 IU/mL of IFN-α. The DeadEnd Colorimetric TUNEL System marks the fragmented DNA in situ with biotinylated nucleotides by terminal transferase rTdT. The complex was detected with streptavidin-horseradish peroxidase (HRP) using the chromogen substrate diaminobenzidine and H2O2. As a result of the procedure the nuclei are stained dark brown.

To determine the effect of V proteins on nuclear DNA fragmentation, TUNEL colorimetric assay (Terminal deoxynucleotidyl transferase Biotin-dUTP Nick End Labeling) was performed in cells expressing the V proteins, which were treated with IFN-α. In cells positive for apoptosis, nuclei are stained brown. Color RGB-type images were obtained using a Nikon microscope (Nikon Inc., Mel, NY) coupled to a Cyber-Shot DSC-W55 (Sony). The images were processed using the ImageJ program (NIH) through its conversion to the type-8 bit and the Analyze Particles tool with a size of 248 × 201 pixels, and circularity of 0.5-1.0, excluding the edges. In cells without transfection and IFN treatment, the rate of apoptotic cells was <3%, whereas there was 20% of DNA fragmentation in cells that express the VWT protein and without change with the VGly protein. In cells treated with IFN-α there were TUNEL-positive cells. However, in those cells that express VWT and VGly, DNA fragmentation decreased 85% and 87%, respectively (Figure 2). Despite inhibition observed on nuclear DNA fragmentation by the V proteins of mumps virus in cells treated with IFN, it was necessary to show that with a strong activation of apoptosis such protein could regulate the extent of DNA digestion. The system was treated with ibuprofen (50 μg/mL) for 12 h and then subjected to TUNEL assay. Ibuprofen is a nonsteroidal anti-inflammatory drug that promotes mitochondrial damage by the modification of oxidative phosphorylation, inhibition of ATP production, modification of permeability of inner membrane and opening of mitochondrial permeability transitory pore, and axis of triggering an apoptotic signal [21]. In non-transfected cells of cervical adenocarcinoma, treatment with the compound produced 95-98% of TUNEL-positive cells; however, with expression of VWT and VGly, the number of positive cells was reduced 79% and 74%, respectively. Comparison between cells expressing the V protein showed that nuclear DNA fragmentation is higher in cells with VWT (Figure 2). Our data suggest that V protein of Urabe AM9 may be a protective or modulating factor for apoptosis, as shown for V protein of SV5 and C protein of Sendai virus [19,22]. Moreover, it was interesting to observe that stimulation with IFN-α activates the caspases in our study and that the V proteins of mumps virus decrease its activation. However, although V protein expression promotes activation of the studied caspases, there is induction of apoptosis and, simultaneously, antiapoptotic activity is present when the cellular system was stimulated with IFN-α. This has been observed with other viruses that cause both apoptotic and anti-apoptotic effects through several viral proteins with the aim of regulating the timing of apoptosis, after viral replication but before activation of antiviral defense systems. Such is the case of the V protein of measles virus that inhibits the p73 protein involved in the regulation of apoptosis [23], whereas its protein C interferes with the induction of apoptosis by blocking PKR. With this virus, pro- and anti-apoptotic events occur simultaneously [24]. We should mention that the usual activities of the pro- and anti-apoptotic protein V could be the result of two opposing activities between V and I proteins of mumps virus as with the V and C proteins of Sendai virus. It is likely that the apoptosis inhibitory activity resides in protein V, whereas the process-inducing activity resides in I protein, a fact that is being studied by our group.

**Figure 3:**
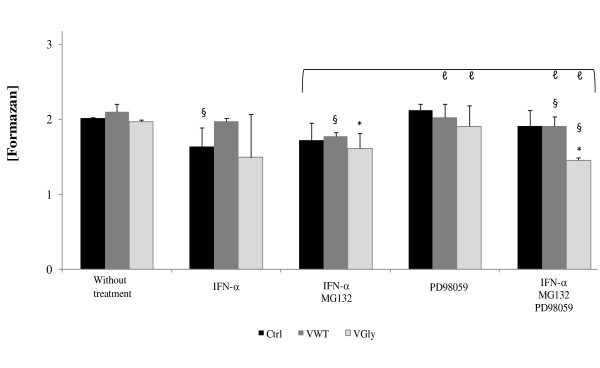
**Proliferation assay in the C33 cell line expressing VWT and VGly proteins of mumps virus**. Proliferation was measured with CellTiter 96 Aqueous One Solution Cell Proliferation Assay (Promega). Cells were treated 12 h with IFN-α, 12 h with MG132 and 2 h with PD98059. The absorbance (490 nm) was measured in a Shimadzu spectrophotometer, 4 h after addition of MTS. Each column represents the mean ± SD of three independent experiments. *, statistical difference between treatment groups and the control group. §, statistical difference between samples with and without treatment. ℓ, statistical difference between the samples treated with PD98059 and triple therapy with respect to samples treated with IFN/MG132. Each column represents the mean ± SD of three independent experiments. Student t-test (*P *< 0.05).

Activation of type-I IFN activates other signal transduction pathways such as the MAPK pathway that regulates several IFN-initiated processes as apoptosis and cell proliferation. A key enzyme in both processes is p38 MAPK that mediates the growth-suppressive effect of type-I IFN, stress signals, inflammatory response that induce apoptosis and is regulated by ERK1/2 [25,26] related to cell proliferation. The MAPK pathway may be a target of mumps virus to control IFN-α-induced apoptosis and regulate cell proliferation. To assess this, we studied the effect of V proteins on proliferation under conditions of antiproliferative stimulus. Determination of cell proliferation was performed with the CellTiter 96 Aqueous One Solution Cell Proliferation Assay (Promega, Madison, WI, USA) in cells treated with IFN-α2b, MG132 and PD98059 (2'-amino-3'-methoxyflavone, a selective inhibitor of MAP kinase kinase 1 and 2 (MEK1/2), enzymes responsible for activating ERK1/2. The quantity of MTS bioreduced to formazan in the assay is measured spectrophotometrically at 490 nm and is directly proportional to the number of living cells in culture. In cells expressing V proteins without chemical stimulus, cell proliferation is not affected (Figure 3). Activation of the IFN pathway by IFN-α2b shows that, in control cells and those cells that express VGly, cell proliferation is decreased 19% and 26%, respectively, in regard to the system without treatment. However, cells with the VWT protein are not affected. The combination of IFN-α2b and MG132 decreases proliferation of the C33 cell line, with or without V proteins, between 14% and 20%, respectively. When PD98059 was used (conditions of non-proliferation) without activating the IFN pathway we did not observe the affectation in cell proliferation with those cells expressing V proteins. Under conditions of three treatments with IFN-α, MG132 and PD98059, it was shown that proliferation decreased only in cells expressing the VGly protein (Figure 3). The VWT protein appears to be potentially protective against apoptotic stimuli such as anti-proliferative IFN-α2b and IFN-α2b, MG132 and PD98059.

Finally, our data provide evidence with regard to the VWT protein of the Urabe AM9 vaccine strain of mumps virus, which seems to exert a best modulatory role on apoptosis and cell proliferation with regard to the VGly protein. This suggests that, in viral pathogenesis, the regulation of apoptosis potentially mediates the damage. The detailed mechanism for the implementation of such activity should be studied in a future investigation. Information of the identity of caspases induced or inhibited by some factor in the mumps virus infection is important in order to establish the molecular mechanism of the virus in the intervention of apoptosis. This knowledge may suggest likely targets for antiviral development.

## Competing interests

The authors declare that they have no competing interests.

## Authors' contributions

NHRM carried out the experimental techniques: nucleic acid purification, cell culture, transfection assays, caspase and TUNEL assays, and cell proliferation and participated in data analysis and in drafting of the manuscript.

GSL participated in the TUNEL assay, data analysis and assisted in drafting the manuscript.

JRL participated in data analysis and assisted in drafting the manuscript.

FSJ participated in the statistical analysis.

IHC participated in cell proliferation assay, data analysis and assisted in drafting the manuscript.

All authors read and approved the final manuscript.
